# ‘There’s a Wall There—And That Wall Is Higher from Our Side’: Drawing on Qualitative Interviews to Improve Indigenous Australians’ Experiences of Dental Health Services

**DOI:** 10.3390/ijerph17186496

**Published:** 2020-09-07

**Authors:** Skye Krichauff, Joanne Hedges, Lisa Jamieson

**Affiliations:** Dental School, University of Adelaide, Adelaide 5005, Australia; skye.krichauff@adelaide.edu.au (S.K.); joanne.hedges@adelaide.edu.au (J.H.)

**Keywords:** Indigenous Australian, qualitative interviews, dental service provision, barriers, enablers

## Abstract

Indigenous Australians experience high levels of untreated dental disease compared to non-Indigenous Australians. We sought to gain insight into barriers that prevent Indigenous Australians from seeking timely and preventive dental care. A qualitative study design was implemented, using face-to-face interviews conducted December 2019 to February 2020. Participants were 20 Indigenous Australians (10 women and 10 men) representing six South Australian Indigenous groups; Ngarrindjeri, Narungga, Kaurna, Ngadjuri, Wiramu, and Adnyamathanha. Age range was middle-aged to elderly. The setting was participants’ homes or workplaces. The main outcome measures were barriers and enablers to accessing timely and appropriate dental care. The findings were broadly grouped into eight domains: (1) fear of dentists; (2) confusion regarding availability of dental services; (3) difficulties making dental appointments; (4) waiting times; (5) attitudes and empathy of dental health service staff; (6) cultural friendliness of dental health service space; (7) availability of public transport and parking costs; and (8) ease of access to dental clinic. The findings indicate that many of the barriers to Indigenous people accessing timely and appropriate dental care may be easily remedied. Cultural competency training enables barriers to timely access and provision of dental care to Indigenous Australians to be addressed. The findings provide important context to better enable health providers and policy makers to put in place appropriate measures to improve Indigenous people’s oral health, and the Indigenous oral health workforce in Australia.

## 1. Introduction

Poor oral health has considerable impact on quality of life and general well-being. Indigenous Australians suffer disproportionately poor oral health relative to non-Indigenous Australians. They have higher levels of untreated dental caries (tooth decay) and periodontal (gum) disease, and are less likely to have received preventive dental care [1]. They tend towards unfavorable dental visiting patterns, broadly associated with accessibility, cost, and a lack of cultural awareness by some service providers [2,3].

In 2009, the Australian government recognized that Aboriginal and/or Torres Strait Islander Peoples (respectfully referred to as Indigenous Australians from hereon) are about 20 per cent less likely to visit a dentist than non-Indigenous Australians and that ‘poor oral health can affect educational and employment outcomes, and can exacerbate other chronic diseases’ [4]. In the 2017–2018 National Survey of Adult Oral Health, higher proportions of Indigenous Australians (compared with non-Indigenous Australians) delayed dental care due to cost, avoided eating certain foods because of dental problems, had experienced toothache in the last 12 months, felt uncomfortable about their dental appearance and perceived a need for dental care [5]. In July 2018, 37 Australian health organizations—including the Australian Dental Council and the Dental Board of Australia—signed the National Registration and Accreditation Scheme Statement of Intent, committing them to achieve equity in health outcomes between Aboriginal and Torres Strait Islander people and other Australians by 2031 [6]. Nonetheless, the compilers of the 2020 Closing the Gap Report clearly state that the National target ‘to close the life expectancy gap by 2031 is not on track’ and that social determinants such as education, employment status, housing and income are responsible for at least 34 per cent of the health gap between Indigenous and non-Indigenous Australians [7].

The literature shows that barriers to health care access and lack of cultural inappropriateness prevent positive outcomes for Indigenous health [8]. To date there have been few examples of evidence exploring the social determinants, providing solutions to barriers or enabling a space for the voices of Indigenous people [9]. This is especially true in the oral health context. One effective method through which to identify entrenched social barriers is through qualitative research [10]. The aim of this study was to thus obtain a greater understanding of people’s perceptions, experiences of oral health, and the barriers and enablers that prevent Indigenous Australians from seeking timely and preventive dental care, using qualitative methodology.

## 2. Methodology

This qualitative project is a nested sub-study of a larger study examining oral human papilloma virus (HPV) infection among Indigenous South Australians [11]. Ethical approval for the nested qualitative sub-study was provided by the University of Adelaide’s Human Research Ethics Committee (HREC-2016-246) and Aboriginal Health Research Ethics Committee (AHREC 14-18-865). All participants provided signed informed consent. The interviewer was a historian with specialized knowledge of South Australian Indigenous people’s colonial and twentieth century history, was sensitive to the intergenerational transmission of trauma and had extensive experience working with Indigenous people of diverse backgrounds and ages. The interviewer spent a lot of time developing a rapport with each interviewee prior to commencing interviews, providing information about the project and answering questions. This was over the course of several days prior to the formal interview and occurred either over the telephone or at an Aboriginal Community Controlled Health Organisation. Interviews were semi-structured, based on a life history approach and drawing from settler colonial theory [12]. The interview questions were developed by the research team in collaboration with the broader study’s Senior Indigenous Research Officer, Indigenous Reference Group, and Aboriginal Health Research Ethics Committee. The aim of the interview guide was to generate questions that would capture, compare and evaluate Indigenous people’s perceptions of their oral health, general health and experiences with the health system across the life course. At some point, all participants were asked about their: (1) earliest memories of caring for their teeth; (2) first visits to the dentist; (3) the health of their teeth throughout their childhood and adulthood; (4) the health of their parents’, grandparents’, children’s, and grandchildren’s teeth; (5) their own and the Indigenous community’s positive and negative experiences with dentists and the health care system; and (6) visions for how Indigenous participants would like to see future oral health and oral health care for their own mob. At all times the study’s Indigenous Reference Group was available to both the interviewer and study participants, as was the parent study’s Senior Indigenous Research Officer (JH).

### 2.1. Setting

Of the 20 completed interviews, six took place in participants’ work places and the remainder in participants’ homes. Eighteen participants resided in metropolitan Adelaide, with two in or near Victor Harbour (an hour and a half south of Adelaide).

### 2.2. Sampling Strategy

Inclusion criteria included identifying as having an Aboriginal and/or Torres Strait Islander background, being aged 18+ years, residing in South Australia and feeling comfortable conducting an interview in English. Exclusion criteria included not identifying as Aboriginal and/or Torres Strait Islander, being aged less than 18 years, not residing in South Australia or not able to conduct the interview in English. Contact details of participants in the parent study who had expressed an interest in being interviewed were provided to the researchers. Recruitment snowballed from initial contacts. On completing their own interview, several participants contacted relatives and friends and provided the oral historian with contact details of others interested in being interviewed.

### 2.3. Data Collection Techniques

Interviews were recorded, with participant consent, on a digital audio recorder and professionally transcribed. Interview length ranged from 20 min to 1 h 10 min; the average length was 36 min. The gender balance was equal: ten women and ten men. Six different South Australian Indigenous groups were represented (Ngarrindjeri, Narungga, Kaurna, Ngadjuri, Wiramu, Adnyamathanha), as well as central Arrente and Torres Strait Islander peoples. Backgrounds were diverse: seven participants spent some of their childhood on missions/reserves (four at Raukkan, two at Point Pearce, one at Ernabella) and 10 participants grew up in or spent substantial portions of their childhood in Adelaide (including two at Colebrook Home, an institution for Australian Indigenous children run by the United Aborigines Mission that was sited at Eden Hills (just outside Adelaide) from 1944 to 1972). With the exception of one interviewee who was fostered at six months and adopted into a white family, all came from large extended families and, implicitly or explicitly, expressed the significance of their family to their sense of identity and well-being. Occupations included health educators, cultural educators, youth workers and careers in Indigenous affairs and advocacy.

### 2.4. Analysis

Data saturation was reached after 20 interviews. A two-stage approach to coding was used in the thematic analysis of transcripts from the interviews. A primary analytical framework (the first stage) was structured using the interview guide to create a coding scheme [13,14]. Grouping of responses occurred according to themes of what was similar and what was different, and was categorized according to questions asked from the interview script. Developing thematic codes was the 2nd step, which occurred in the next stage of analysis. The a priori conceptual frame upon which analyses were conducted was based on settler colonial theory [15]. This phase of analysis sought to more deeply reveal the assumptions, perceptions and conceptual constructs that undergirded participants’ perceptions of their own oral health, general health, and experiences with the health system across the life course. Names used within the paper are aliases. Each participant was provided with a transcript of their interview to ensure what had been recorded was a true and accurate record of what the participant felt had been discussed.

The objective was to demonstrate the sustained and entrenched impacts of settler colonialism on Indigenous perceptions of their health and wellbeing through an oral health lens. For teeth, and the health of teeth, provide a unique insight into the structural and historical factors that contribute to the health gap between Indigenous and non-Indigenous Australians. This is because oral health provides a contemporary and simultaneous reflection of social determinants (dental diseases are socially patterned) [16] and inequities in access to dental services (restored teeth versus teeth with untreated decay) across the life span. All three of the authors were involved in the analysis, with the study’s Senior Indigenous Research Officer providing insights in the interpretation of the findings based on her first-hand cultural knowledge and lived experience.

### 2.5. Nature of the Engagement, Involvement and Leadership by Indigenous People

Participants in this project were part of the broader study examining oral HPV infection among Indigenous South Australians. This broader study is governed by an Indigenous Reference Group (IRG), comprising Indigenous community members, councilors, and health workers, and is chaired by an Indigenous health manager. The IRG provides oversight and cultural guidance on all aspects of the study, including recruitment strategies and data collection. The study team is led by a senior Indigenous research officer (JH), who has published more specific details of the Indigenous engagement processes implemented in the study [17].

### 2.6. Whose Priorities are Being Addressed by the Study

Priorities of the participants are being addressed by the study, with the idea for the work arising from much anecdotal feedback provided by other Indigenous community members during past and present research interactions. Because most policy will not be informed without ‘evidence’, it was the decision of the Indigenous reference group (who were involved in both the study’s conception and design) and study authors to empirically investigate the research question and to publish the findings.

## 3. Results

Age, gender, place of birth, places of residence, family history, childhood, adolescent and adult experiences, education level, and occupation were all constructs which influenced the emerging themes. The findings emphasized here focus primarily on the barriers and enablers participants referred to with respect to access timely dental care.

### 3.1. Childhood Experiences

Approximately 66% of participants did not recall seeing a dentist when they were young. About 75% did not think they used a toothbrush or toothpaste throughout their childhoods. Several explicitly stated that the health of their teeth was not a priority. Cheryl, a 62 year old, grew up on the West Coast of South Australia. Cheryl said that when she moved to Adelaide in her early twenties, ‘*I didn’t know, didn’t know much about teeth*’. When she was young, teeth weren’t an issue, ‘*it was like, making sure you got something to eat*’. Ned, also 62, grew up in a shack on the Coorong (stretch of coast south of Adelaide). He ‘*never saw a toothbrush’* and was sure his brothers ‘*would never have picked up a toothbrush ever*.’ Ned doesn’t remember going to the dentist as a kid as ‘*it just wasn’t a priority’*—‘*All we thought about was having a good feed*’.

Anthony, a 49 year-old, works as an Indigenous Outreach Worker helping Indigenous peoples manage their own health; ‘*we go and talk [to people] … who’s your GP, (pause) when was the last care plan done … we even talk dental, you know, when’s the last time you seen the dentist, when’s the last time you had your eyes tested’.* Anthony said most of his clients do not visit dentists and he provided several reasons why. High on his list was ‘*fear of the going to the dentist*’. His observation tallied with comments made by virtually all participants. When asked about their first dental visit, participants remembered being afraid and the pain of being injected with ‘*big needles*’ and when having teeth pulled. In Ned’s words, ‘*the one thing that scares people is the needle …that’s why they avoid the place, they know they’re going to get drilled, they know—that’s why they’d rather let the teeth rot (pause) and then get it pulled out*’. Early memories of fear and pain remain vivid throughout participants’ lifetimes despite more positive recent experiences. Participants stated or implied they only go to the dentist if there is a problem. Thirteen participants had dentures or plates and so did not see the need to visit a dentist—two people had not been back to a dentist since receiving dentures over thirty years ago. In the words of 64 year-old participant Alex, ‘*They’ll only come in if they’ve got a toothache or something*’.

### 3.2. Communication Barrier

Challenges surrounding communication/language were noted as a substantial barrier to making and attending appointments. This was demonstrated by Anthony, who said a key reason why his clients didn’t visit dentists was ‘*a communication barrier’* and referred to ‘*ringing, making that appointment*’. A critical part of Anthony’s job is to ‘*make the phone call for them*’. Sometimes Anthony has been put on hold for 45 min when making an appointment. Long waiting times on the telephone are frustrating and, when using a pre-paid mobile phone, expensive. They also use up battery charge. As 77-year-old Yvonne pointed out, ‘*even when they [Indigenous people] ring up to make an appointment, if they don’t answer that phone, they’re not going to bother ringing back no more … I find with our people they don’t have sort of time for it—people don’t answer their phones they’re not going to ring back, even with doctors or whatever*’.

The same applies to follow-up appointments. Alex recalled that the dentist at an Aboriginal Community Controlled Health Organisation in Adelaide said he’d make another appointment for Alex, but ‘*he never rang back*’. Alex said ‘*I’m not worrying about him if he’s not going to worry about me. When he says something, you’re gonna do something and (pause) cause I’m thinking … that’s probably how he treats other black fellas, he knows they’re not gonna come back—“if I don’t ring them, they won’t come”*’.

Stephanie, aged 69, bluntly, although not unsympathetically, described her people’s reluctance to make appointments; ‘*You know you get some dumb-bells that don’t, oh—they’ve got an excuse, so what they do is they get back further and further with the appointments … And then, they’re idiots then they realise “Oh well, I should’ve rang up”. I say “Well they should’ve”*’. The desire for clinic staff to be proactive was reiterated by Roy, a 42 year old Torres Strait Islander, who said ‘*if someone rings up [for an appointment], ring them back’, otherwise ‘months go past and you kind of like, “oh, well it’s not hurting so, just move on”, but I think, yeah, ring people back*’. Anthony felt that short waiting times on the phone would make making that appointment much easier. Yvonne referred to the frustration of long waiting times once the appointment was made, noting it ‘*can take 6–12 months to see someone*’. Roy said that health care workers shouldn’t ‘*make people feel guilty for missed appointments*’.

### 3.3. Atmosphere of Clinic

A critical element of Anthony’s role is to accompany people to their health related appointments. He and his co-workers have found ‘*there’s a lot of GP’s that are out there and some surgeries which aren’t really culturally appropriate … and don’t have that respect*’. In clarifying what he meant by ‘culturally appropriate’, Anthony referred to ‘*both the physical space and the attitude of staff*’.

### 3.4. Physical Environment

Anthony gave a new teaching dental clinic as an example saying ‘*it’s not really an Indigenous friendly place*’. Anthony suggested the space could have Indigenous artwork, or an acknowledgement on the wall, the Sorry speech or the Aboriginal flag as they ‘*help put Aboriginal people at* ease’. Phyllis, 74 years, thought having Indigenous people on posters providing information was ‘really good’ and makes ‘a h*** of a difference’.

### 3.5. Perception of Empathy

The empathy of dental staff is crucial to Indigenous people’s experience in health care settings and strongly influences whether Indigenous peoples will return to a clinic. Nora didn’t like an orthodontist her son was seeing because she sensed ‘*it was all about the money, he was just a number, so [we] didn’t go back*’. According to Anthony, lots of Indigenous people don’t like the way dentists talk to them. Cheryl said she saw a ‘*lady dentist [at another Aboriginal Community Controlled Health Organistion] and it’s like, they don’t treat you like you’re human. They talk down to you*’. Earlier experiences caused Cheryl to deduce that people who ‘*are working for low-income earners and Aboriginal organisations (pause), they’re not there with passion, or heart*’.

Several participants made it clear that a good dentist will clearly explain everything they have to do and not just tell their patient what they’re doing, but why. Where possible, they will involve the patient in the decision-making process. Referring to her relatives elsewhere in South Australia, Cheryl said patients need to be shown how to clean teeth and have it clearly explained to them why it is important to clean. (Recently, when Cheryl went to the new University of Adelaide Dental Hospital, a female dentist explained—verbally and through illustrations—everything clearly. Cheryl was surprised, she told the dentist ‘*you’re the first dentist that—oh no, sorry, the second dentist, that I’ve ever been to in my life that’s actually treated me as a human being’*).

### 3.6. Expense and Confusion over Available Services

For participants who were not on a pension or Centrelink, the cost of seeing a dentist is prohibitive. Anthony said it is ‘*just money’* that stops him going. Nora said ‘*You’re stuck between a rock and hard place if you’re not on Centrelink’* and *‘it’s the cost of things that is prohibitive*’. Nora thought it would be good if dental care could be a free service or like Medicare. Significant confusion was evident among participants regarding the Australian Government’s Closing the Gap initiative and services available through the scheme. Worryingly, some participants had never heard of Closing the Gap. Others thought it was no longer funded, others thought dental care was not included. Expressing his frustration with Closing the Gap providers, Ned said ‘*the left hand doesn’t know what the right hand’s doing, you need to get that part in order, because nothing worse than mucking a person around, you know because they’ve got to make other arrangements*’.

### 3.7. Transport/Access to Building

When speaking about the new Dental Hospital, Yvonne said that many of her people can’t afford the parking, and that access to the building is difficult for those using public transport due to the steep decline from the bus stop to the entrance, which is ‘*tricky if you have a walker*’. Anthony similarly commented that the new Dental Hospital is ‘*pretty challenging to get to*’. For Alex, who lives in one of Adelaide’s outer suburbs, ‘*It’s a long way to go into Adelaide to see a dentist*.’ Fred has been battling cancer and has had to attend many appointments but ‘How the h*** do I arrange transport?’

## 4. Discussion

Our findings illustrate the challenges that Indigenous Australians face in accessing culturally safe, appropriate and timely dental care. Many of the barriers identified can be readily ameliorated. These include improving the cultural friendliness in dental health provider spaces, making cultural competency training a regular requirement of all dental health personnel (from receptionists to dental providers to transport officers), and improving childhood experiences of dental care that in turn reduce dental fear in older age [18]. For example, evidence suggests that addressing any fear children may have in the dental setting markedly improves attitudes and attendance for dental care later in the life course [19]. Simple steps can be taken to make Indigenous people feel welcomed. These include: (1) clearly displaying signs (posters, flags, acknowledgment of Country) which positively recognize Indigenous people; (2) demonstrating an awareness and sensitivity towards Indigenous people’s connection to Country and the injustice of the colonial past; (3) educating all health workers (including receptionists, dentists, nurses etc.) of the importance of empathy; (4) explaining procedures thoroughly and, where possible, using visual aids; and (5) including patients in the decision-making process to nurture a sense of autonomy over dental care-related proceedings [18].

Other improvements require greater resources/organizational change. For example, having the funds to answer the phone without putting people on hold for long periods and returning phone calls; persisting with making and chasing up follow-up appointments; not penalizing people who can’t make or forget appointments (instead, it’s important to understand the reasons why and be empathetic); checking people are able to get to appointments and if difficult, jointly problem-solving to come up with solutions. The quote around “*he never rang back*” goes beyond communication barriers, however, and into the importance of trust/integrity and delivering on a commitment. The literature provides evidence of how following up on due diligence regarding Indigenous patient appointments does increase utilization of health services [8]. It is also crucial to have more Indigenous dental staff, with none of the participants mentioning Indigenous staff members in the dental clinics they had attended. Part of the recommendations in a 2019 report from the Australian Medical Association was to dramatically increase the proportion of oral health personnel who identify as Indigenous [18].

Fear was described by numerous participants as a critical reason for dental non-attendance. This is consistent with literature from other vulnerable populations [20] and the general public [21]. Using minimally invasive dental care practices among children has been shown to mitigate the impacts of dental fear in this age group [22], but there is little evidence of what might be effective among adults. Certainly, our recommendations to take the time to explain procedures properly and to ensure understanding of why regular check-ups are important would go some way to alleviating dental fear among all who are dentally anxious.

The institutional racism contributing to much of the participants’ reluctance to attend for dental care requires a whole of society shift [23]. Certainly, the recent Black Lives Matter movement has raised awareness of this, as has the increased profile of many prominent Indigenous Australian leaders. The role of racism in oral health inequalities has been documented at a global level [23]. Although there is limited evidence of successful initiatives to address this, there are some encouraging results from the general health arena in this space [24].

Many participants demonstrated both considerable empathy for, and deep understanding of, the experiences across the lifecourse that impact on Indigenous health and wellbeing. There was universal condemnation of racism in all its forms; overt and covert, personal and institutional. Racism, culture and difference was described by almost all participants. This demonstrates the crucial gap between the critical and conscious requirement of dental health service staff (and policy makers responsible for funding dental health care) to understand how past settler colonial structures of decision making and power have led to their now privileged positions in broader Australian society, and the study participants’ understanding of these issues. In the context of our study, most participants were unable to fully appreciate just how manifestly unjust the power differential is in Australia, with just one outcome of this being the inequitable and unfair models of dental service provision for Indigenous Australians. This poor indictment of both policy makers and health providers means that the need for more content relating to Indigenous health in the training courses of all dental programs in Australia will likely not be acted upon, which has downstream consequences for ongoing racism/non-Indigenous Australian dominance in future provision of dental services.

Limitations of the study include: (1) a non-Indigenous person conducting the interviews; (2) participants from only South Australia involved, whose views might differ in meaningful ways to Indigenous persons in other parts of Australia; and (3) participants were generally in the mainstream; not, for example, in prisons, residential care facilities or homeless shelters. This means the views of these more vulnerable groups were not collected.

## 5. Conclusions

In conclusion, barriers preventing timely access to dental services for Indigenous Australians include (1) fear of dentists; (2) confusion regarding availability of dental services; (3) difficulties making dental appointments; (4) waiting times; (5) attitudes and empathy of dental health service staff; (6) cultural friendliness of dental health service space; (7) availability of public transport and parking costs; and (8) ease of access to dental clinic. Many of the barriers to Indigenous people accessing timely and appropriate dental care may be easily remedied. The findings provide important context to better enable health providers and policy makers to put in place appropriate measures to improve Indigenous people’s oral health. Indigenous peoples have been substantially impacted by racism and settler colonialism [25,26], meaning it is crucial that learning spaces are created in dental and other health programs throughout Australia to enable dialogue so that these insights can be expanded upon and addressed.

## References

[1] Australian Health Ministers Advisory Council (2017). Aboriginal and Torres Strait Islander Health Performance Framework 2017 Report.

[2] Council of Australian Governments Health Council (2015). Healthy Mouths, Healthy Lives: Australia’s National Oral Health Plan 2015–2024.

[3] National Advisory Council on Dental Health (2012). Report of the National Advisory Council on Dent. Health 2012.

[4] Australian Government, Department of Social Services. https://www.dss.gov.au/about-the-department/publications-articles/corporate-publications/budget-and-additional-estimates-statements/closing-the-gap-indigenous-dental-services-in-rural-and-regional-areas.

[5] Australian Research Centre for Population Oral Health (2019). Australia’s Oral Health: National Study of Adult Oral Health 2017–2018.

[6] Dental Board: Australian Health Practitioner Regulation Agency Closing the Gap by 2031: A Shared Commitment. https://www.dentalboard.gov.au/News/2018-07-04-closing-the-gap-by-2031-a-shared-commitment.aspx.

[7] Australian Government (2020). Closing the Gap Report 2020.

[8] Davy C., Harfield S., McArthur A., Munn Z., Brown A. (2016). Access to primary health care services for Indigenous peoples: A framework synthesis. Int. J. Equity Health.

[9] Davidson P., MacIsaac A., Cameron J., Jeremy R., Mahar L., Anderson I. (2012). Problems, solutions and actions: Addressing barriers in acute hospital care for indigenous Australians and New Zealanders. Heart Lung Circ..

[10] Kitto S., Chesters J., Grbich C. (2008). Quality in qualitative research. Med. J. Aust..

[11] Jamieson L., Garvey G., Hedges J., Mitchell A., Dunbar T., Leane C., Hill I., Warren K., Brown A., Ju X. (2018). Human papillomavirus and oropharyngeal cancer among indigenous Australians: Protocol for a prevalence study of oral-related human papillomavirus and cost-effectiveness of prevention. JMIR Res. Protoc..

[12] Veracini L. (2010). Settler Colonialism.

[13] Charmaz K. (2006). Constructing Grounded Theory: A Practical Guide through Qualitative Analysis.

[14] Ryan G.W., Bernard H.R. (2003). Techniques to identify themes. Field Methods.

[15] Veracini L. (2011). Introduction. Settl. Colonial Stud..

[16] Peres M.A., Macpherson L.M.D., Weyant R.J., Daly B., Venturelli R., Mathur M.R., Listl S., Celeste R.K., Guarnizo-Herreño C.C., Kearns C. (2019). Oral diseases: A global public health challenge. Lancet.

[17] Hedges J., Garvey G., Dodd Z. (2020). Engaging with Indigenous Australian communities for a human papilloma virus and oropharyngeal cancer project; use of the CONSIDER statement. BMC Med. Res. Methodol..

[18] Australian Medical Association (2019). 2019 AMA Report Card on Indigenous Health No More Decay: Addressing the Oral Health Needs of Aboriginal and Torres Strait Islander Australians.

[19] Seligman L.D., Hovey J.D., Chacon K., Ollendick T.H. (2017). Dental anxiety: An understudied problem in youth. Clin. Psychol. Rev..

[20] Mago A., MacEntee M.I., Brondani M., Frankish J. (2018). Anxiety and anger of homeless people coping with dental care. Community Dent. Oral Epidemiol..

[21] Armfield J.M. (2011). Australian population norms for the Index of Dental Anxiety and Fear (IDAF-4C). Aust. Dent. J..

[22] Arrow P., Klobas E. (2017). Minimal intervention dentistry for early childhood caries and child dental anxiety: A randomized controlled trial. Aust. Dent. J..

[23] Bastos J.L., Celeste R.K., Paradies Y.C. (2018). Racial inequalities in oral health. J. Dent. Res..

[24] Hunter New England Health Aboriginal and Torres Strait Islander Strategic Leadership Committee (2012). Closing the gap in a regional health service in NSW: A multi-strategic approach to addressing individual and institutional racism. NSW Public Health Bull..

[25] Allan B., Smylie J. (2015). First Peoples, Second Class Treatment: The Role of Racism in the Health and Well-Being of Indigenous Peoples in Canada.

[26] Paradies Y., Ben J., Denson N., Elias A., Priest N., Pieterse A., Gupta A., Kelaher M., Gee G. (2015). Racism as a determinant of health: A systematic review and meta-analysis. PLoS ONE.

